# Swedish snuff and incidence of cardiovascular disease. A population-based cohort study

**DOI:** 10.1186/1471-2261-9-21

**Published:** 2009-05-27

**Authors:** Ellis Janzon, Bo Hedblad

**Affiliations:** 1 Department of Health and Society, Malmö University, Malmö, Sweden; 2 Department of Clinical Science, Research Groups of Cardiovascular Epidemiology Lund University, Malmö University Hospital, Malmö, Sweden

## Abstract

**Background:**

The relationship between smoking and an increased incidence of cardiovascular diseases is well known. Whether smokeless tobacco (snuff) is related to myocardial infarction (MI) or stroke is still controversial. Aim of this study was to explore whether snuff users have an increased incidence of MI or stroke.

**Methods:**

A total of 16 754 women and 10 473 men (aged 45–73 years), without history of cardiovascular disease (CVD), belonging to the population-based "Malmö Diet and Cancer" study were examined. Incidence of MI and stroke were monitored over 10.3 years.

**Results:**

Snuff was used by 737 (7.0%) men and 75 (0.4%) women, respectively. Among men, snuff was significantly associated with low occupation level, single civil status, high BMI and with current and former smoking. In women, snuff was associated with lower systolic blood pressure. A total of 964 individuals (3.5%), i.e.544 men (5.3%) and 420 (2.5%) women suffered a MI during the follow-up period. The corresponding numbers of incident stroke cases were 1048, i.e. 553 men (5.3%) and 495 (3.0%) women, respectively. Snuff was not associated with any statistically significant increased risk of MI or stroke in men or women. The relative risks (RR) in male snuff users compared to non-users were 1.05 (95% confidence interval (CI): 0.8–1.4, p = 0.740) for incident MI and 0.97 (0.7–1.4, p = 0.878) for stroke, after taking age and potential confounders into account. In women none of the 420 (2.5%) women who were snuff users had a MI and only one suffered a stroke during the follow-up.

**Conclusion:**

Several life-style risk factors were more prevalent in snuff-users than in non-users. However, the present study does not support any relationship between snuff and incidence of cardiovascular disease in men.

## Background

The relationship between smoking and an increased incidence of cardiovascular diseases (CVD) is well known. Whether smokeless tobacco, in this study defined as snuff, is related to incidence of CVD is less clear. Lately several studies have explored the relationships between snuff and CVD, and the results are not consistent [1-13]. In an earlier study of construction workers, a significantly increased risk of MI was reported among snuff users [7,8]. Snuff users also had a significantly higher prevalence of hypertension in that study [8]. In contrast, several other studies from the general population found no relationship between snuff and incidence of MI [2-5,10,12,13]. Some scientists have highlighted the need for more studies on snuff and incidence of CVD[1,2]. The purpose of this population based cohort study was to explore whether snuff users have an increased incidence of myocardial infarction (MI) and stroke.

## Methods

### Subjects

The Malmö Diet and Cancer (MDC) cohort was used to examine snuff use and its effect on incidence of CVD. All subjects who were 45–73 years old, and who lived in the city of Malmö, Sweden were invited. The health examination was performed at the Malmö University Hospital from March 1991 to February 1996. The subjects were invited by letters and advertisement in newspapers. Of the eligible population of approximately 70 000 citizens, 28 449 subjects, i.e. 17 203 women and 11 246 men, respectively participated. The participation rate was approximately 40% [14,15]. The MDC cohort and the characteristics of the non-participants have been described previously [15]. The representativeness of the MDC study has been evaluated by a comparison with subjects, in corresponding age groups, who participated in 1994 in a mailed questionnaire health survey in the city of Malmö [15,16]. In that survey, non attendance was associated with a lower prevalence of smoking and a higher rate of mortality. However, prevalence of smoking and obesity in the MDC was rather similar compared to the mailed health study (prevalence of smoking 39.9% vs.37.5% and 12.4% vs. 11.8% for prevalence of obesity, respectively).

After exclusion of subjects with a history of MI or stroke, and subjects with missing information about BMI, blood pressure, diabetes or tobacco habits, the cohort consisted of 27 227 individuals, 16 754 women and 10 473 men, respectively. Mean age was 57.4 +7.6 years in women and 59.1+7.0 years in men.

### Screening examination

Measurements of height (in cm), weight (in kilograms) and blood pressure (in mmHg) were performed. The participants also completed a comprehensive self-administered questionnaire about life style habits and medical history and treatment [14].

### Smoking habits and use of snuff

Smoking was assessed in a self-administered questionnaire, and the subjects were categorized as never smokers, ex-smokers and current smokers (regular or occasional smokers). Daily cigarette consumption among current smokers was assessed by the questions "how many cigarettes do you smoke per day"?

Snuff use was assessed by the question "Do you use snuff?" In subjects confirming the use of snuff the weekly consumption of snuff was obtained by the question "How many packages of snuff do you consume every week?" We dichotomized weekly snuff consumption into three groups: a) 1–2 packages per week as low consumption, b) 3–5 packages per week as medium consumption and c) more than six packages per week as high consumption.

### Chewing tobacco and use of nicotine gum

The habits of tobacco chewing and using nicotine gum were obtained by the questions: ""Do you chew tobacco" and "Do you use nicotine chewing gum?"

### Hypertension

Blood pressure was measured twice in the supine position after 10 minutes rest using a mercury sphygmomanometer. Subjects who had systolic blood pressure (SBP) _140 Mm/Hg or diastolic blood pressure (DBP) _90 mmHg or blood pressure-lowering drug treatment of hypertension were considered to have hypertension [17].

### Diabetes mellitus

Participants who reported that they had diabetes or who used anti-diabetic medication was considered to have diabetes. As fasting blood glucose and lipids were available only on subjects belonging to the cardiovascular project (n = 5 500) [18], It was not possible to define diabetes on all study subjects according to present or previous recommendations for classification of diabetes mellitus [19].

### Body mass index (BMI)

BMI was calculated as weight/height^2 ^(kg/m^2^).

### Physical activity

Physical activity during leisure time was revealed through 18 questions covering a range of activities in the four seasons. The number of minutes per week for each activity was multiplied by an intensity coefficient, and an overall leisure time physical activity scores was created. Scores were divided into four quartiles and subsequently categorized as a) low (quartile 1), b) moderate (quartile 2 to 3), and c) high (quartile 4) levels [20].

### Information on socio-economic circumstances

Information on occupation and marital status was assessed by the questionnaire. The categorization into occupational groups based on the Swedish socio-economic index (SEI) [21] has been presented previously [22,23]. This classification takes into consideration the educational level needed for the job, the level of responsibility in the work organization as well as the actual work task [22]. In this study data were dichotomized into four groups only, due to few cases. High and medium levels non-manual employees was dichotomized into the same group as "High occupational level" (SEI groups 46–69)", and contained the following groups: high level (i.e., business executives, university-engineers and teachers) and medium level employees (i.e., reg. nurses, employees, computer operators and high school teachers).

Low-level non-manual employees (SEI groups 33–36, i.e. office assistants, sales staff and secretaries, etc) were categorized as "Medium occupation level".

Skilled manual workers, unskilled workers and unspecified occupational groups (i.e. early retired women, housewives, students and unemployed) (SEI groups 11–22) were categorized as "low level occupational". The group "others" contained self employed* and farmers i.e. (SEI groups 70–99) [22,23].

### Marital status

The questionnaire had the following four categories: Married, unmarried, divorced and widowed. In accordance with previous studies we dichotomized the cohort into the status living alone or not [22].

### Education

Education level was assessed in the questionnaire as previously described and divided into low (9 years education or less) and high education level (at least secondary graduation). The dichotomized variable education was used in the analyses [22].

### Incidence of stroke and MI

Each participant was followed from the baseline examination until the first incident MI or stroke event, death, emigration out of Sweden, or Dec 31, 2004, whichever came first.

Incident stroke was defined as ICD-9 codes 430, 431, 434 and 436 [24,25]. MI was defined as nonfatal MI (ICD-9 code 410) or fatal ischemic heart disease (ICD-9 code 410–414). The National hospital discharge register, the Stroke register of Malmö [26] and the Swedish hospital discharge register were used for case retrieval.

### Statistics

The Cox proportional hazards model was used to assess the relationship between snuff use and incidence of MI and stroke, respectively. The Cox model was also used to adjust the relationships for other risk factors. One way analyses of variance and logistic regression was used to compare risk factor in users and non-users of snuff. A general linear model was used to adjust the mean values (i.e., blood pressure and BMI) for other risk factors

## Results

Snuff was used by a total of 812 individuals, i.e. 737 (7.0%) men and 75 (0.4%) women, respectively. Of those subjects 764 (94.1%) reported weekly snuff packages consumption. Among men 54.5% (n = 402) were categorized as low, 32.2% (n = 237) as medium and 9.0% (n = 66) as high consumers. The corresponding figures among women were 58.7% (n = 44), 14.7% (n = 11) and 0.1% (n = 4), respectively.

Chewing tobacco was unusual in this cohort, being reported by only 96 persons (0.4%). Nicotine gum was used by a total of 497 individuals (1.8%), i.e. 131 (26.4%) men and 364 (73.6%) women, respectively data not presented in tables.

### Snuff, smoking habits and cardiovascular risk factors

The distribution of risk factors in users and non-users of snuff is presented in Table 1. Mean age for snuff users was significantly lower in both men and women compared to non-users. Smoking and former smoking was more common in snuff-users compared to non-users. However, the mean daily cigarette consumption (in grams) in snuff users compared to non-users was significantly lower both among male and female current smokers. Former smoking was also higher among snuff-users compared to non-users, significantly only for men. Very few never smokers reported use of snuff (i.e. 67 men and 27 women). BMI was slightly higher in male snuff users, and this relationship remained significant after adjustment for age [adjusted means ± standard error of the mean; 26.2 ± 0.035 vs. 26.6 ± 0.013 kg/m^2^]. In men, blood pressure was rather similar in snuff users and non-users, however, prevalence of blood pressure lowering treatment was significantly lower in male snuff-users (i.e. 14.9% vs. 18.5%, p = 0.016) [Table 1]. Female snuff users had statistically significant lower systolic blood pressure and prevalence of hypertension than non-users. These relationships were markedly attenuated and became non-significant after taking age and BMI into account (data not shown) In addition to that, fewer male snuff users than non-users were currently on treatment with blood pressure lowering agents (i.e. 14.9% vs. 18.5%, p = 0.016 for men, and 9.3% vs. 15.7%, p = 0.129 for women [Table 1].

**Table 1:** Distributions of risk factors in 27 227 individuals in relation to smokeless tobacco (snuff)

	Men (n = 10 473)			Women (n = 16 754)		
	
	Snuff non users (n = 9 736)	Snuff users (n = 737)	P value	Snuff non users (n = 16 679)	Snuff users (n = 75)	P value
Age (years)	59.2 ± 7.6	56.8 ± 6.7	<0.001	57.4 ± 7.9	54.6 ± 7.2	0.002
Blood pressure (BP, mm Hg)						
Systolic	143.9 ± 19.3	143.0 ± 19.3	0.227	139.2 ± 20.2	134.4 ± 21.2	0.040
Diastolic	88.0 ± 9.8	88.2 ± 9.9	0.587	84.0 ± 9.7	82.0 ± 10.4	0.090
Hypertension, % (n)	65.2 (6351)	65.8 (485)	0.708	53.2 (8872)	37.3 (28)	0.006
Use of BP lowering treatment, % (n)		14.9 (110)	0.016	15.7 (2623)	9.3 (7)	0.129
BMI (kg/m2)	26.2 ± 3.5	26.6 ± 3.5	0.004	25.4 ± 4.2	25.1 ± 3.9	0.528
Diabetes mellitus %(n)	3.9 (368)	3.9 (29)	0.832	2.4 (398)	1.3 (1)	0.551
Smoking habits						
Never smoker, % (n)	30.3 (2946)	9.1 (67)	0.000	44.4 (7404)	36.0 (27)	0.144
Ex-smoker, % (n)	41.2 (4014)	57.0 (420)	0.000	27.6 (4599)	36.0 (27)	0.103
Current smoker,%(n)	28.5 (2776)	33.9 (250)	0.002	28.0 (4676)	28.0 (21)	0.995
Daily (grs) cigarette consumption by current smokers	16.1 ± 9.0	12.3 ± 9.7	0.001	12.9 ± 7.1	7.8 ± 6.4	0.001
Occupational status						
Low level, % (n)	45.4 (3866)	57.0 (369)	0.000	44.3 (6522)	25.2 (25)	0.122
Medium level, % (n)	18.0 (1532)	13.8 (89)	0.007	20.0 (2944)	23.9 (17)	0.409
High level, % (n)	14.5 (1234)	11.3 (73)	0.025	14.2 (2092)	22.5 (16)	0.460
Others, % (n)	22.2 (1889)	17.9 (116)	0.212	21.4 (3151)	18.3 (13)	0.524
Civil status						
Single, % (n)	27.0 (2630)	32.6 (240)	0.001	39.2 (6545)	46.7 (35)	0.117

Values are shown as mean ± SD unless otherwise stated. For occupational status percentages are of numbers classifiable

### Snuff and socio-demographic risk factors

Significantly more male snuff users than non-users were living alone and categorized as belonging to the low-level occupational group. Among women, however, the proportion that was categorized as belonging to low-level occupational group was lower among snuff users compared to non-users 25.2% vs. 44.3%, p= 0.122 [Table 1].

### Incidence of cardiovascular disease

During the follow-up period a total of 544 (5.3%) men had MI and 553 (5.3%) had stroke. The corresponding figures for women were 420 (2.5%) for MI and 495 (3.0%) for stroke (data not shown I tables).

In men, snuff use was not significantly related to risk of incident MI during the follow-up period, whether adjustment was made for age only (relative risk, RR = 0.89, 95% confidence interval CI: 0.7–1.2), or when taking other risk factors into account (RR: 1.05; 95% CI: 0.8–1.4 [Table 2].

**Table 2:** Incidence of and RR (95% CI) for MI and stroke in relation to snuff in men

		**Smokeless tobacco (snuff)**		
		
		**No **N = 9736	**Yes **N = 737	*P value*
**MI n (%)**	All	511 (5.2)	33 (4.5)	
	Never smokers	114 (3.9)	4 (6.0)	
	Ex-smokers	180 (4.5)	15 (3.6)	
	Smokers	217 (7.8)	14 (5.6)	
Age-adjusted RR	All	1.0	0.89 (0.7–1.2)	0.408
	Never smokers	1.0	0.61 (0.2–1.5)	0.280
	Ex-smokers	1.0	0.79 (0.5–1.2)	0.251
	Smokers	1.0	1.2 (0.8–1.9)	0.393
Risk factor adjusted*	All	1.0	1.05 (0.8–1.4)	0.740
	Never smokers	1.0	0.75 (0.3–1.8)	0.532
	Ex-smokers	1.0	0.81 (0.5–1.2)	0.304
	Smokers	1.0	1.31 (0.8–2.0)	0.236
**Stroke n (%)**	All	518 (5.3)	35 (3.5)	
	Never smokers	136 (4.6)	4 (6.0)	
	Ex-smokers	211 (5.3)	18 (4.3)	
	Smokers	171 (6.2)	13 (5.2)	
Age-adjusted RR	All	1.0	0.88 (0.6–1.2)	0.470
	Never smokers	1.0	0.54 (0.2–1.8)	0.226
	Ex-smokers	1.0	0.91 (0.6–1.5)	0.719
	Smokers	1.0	1.03 (0.6–1.8)	0.929
Risk factor adjusted*	All	1.0	0.97 (0.7–1.4)	0.878
	Never smokers	1.0	0.59 (0.2–1.5)	0.311
	Ex-smokers	1.0	0.88 (0.5–1.4)	0.632
	Smokers	1.0	1.13 (0.6–2.0)	0.663

* adjusted for age, BMI, smoking habits, diabetes mellitus, hypertension, physical activity, marital status and occupation, RR, relative risk and 95% confidence interval.

In men who used snuff, the age-adjusted RR for stroke was 0.88, 95% CI: 0.6–1.2. This RR remained quite similar after taking other risk factors into account (RR: 0.97; 95% CI: 0.7–1.4 [Table 2]. No significant relationship between snuff and incidence of MI or stroke was observed in any of the categories of smoking. However, the number of MI and stroke were very small among snuff users who never had smoked (n = 4 and n = 4, respectively).

None of the 420 women who suffered a MI were snuff users. Furthermore, there was only one stroke case among female snuff users. As the number of events was based on only one case no further statistical analysis was performed on this matter among women.

The age-adjusted RR for incident MI was 1.5 times higher (95% CI: 0.5–4.5, p = 0.512) in men who chew tobacco than in men who used snuff. After further adjustment for other risk factors it was even higher (RR: 1.7, 95% CI: 0.5–5.2, p = 0.368, data not shown in tables). The RR for MI in tobacco chewing women was not evaluated due to small numbers.

## Discussion

It is well known, and well accepted, that tobacco smoke has many negative health effects [27,28]. The effects of smokeless tobacco, snuff, on health are less well explored, though lately more studies have been published [1-13,29-31]. Fewer studies have explored the association between snuff and CVD, and to our knowledge, there are only few studies on women [3,10]. During the 10-year follow-up only one of the 75 female snuff users suffered a stroke and none a MI. Because of these small numbers in females, our analyses are restricted to males. The present study does not support the hypothesis that snuff is a risk factor for incident myocardial infarction or stroke in men. We also could not confirm previously reported findings that snuff users have higher blood pressure than non-users [8].

Snuff users generally had lower occupation level, among men, and were more often single and current or former smokers than non-users of snuff. BMI was somewhat higher in male snuff users. There was, however, no indication of any significant relationship between snuff and an increased prevalence of hypertension or diabetes. Mean blood pressure showed no statistically significant difference between male or female snuff users and non-users after taking age and BMI into account. The absence of a relationship between snuff use and high blood pressure is in accordance with results from other studies discussed in a review study by Lee 2007 [5] but in contrast to a study of construction workers [8].

Both smoking and smokeless tobacco have been associated with lower weight [31]. In this study male snuff users had higher BMI than non-users, which remained after adjustment for age and smoking habits. No difference was observed in women. Because snuff often is used by smokers who try to quit, and smoking cessation is associated with weight gain, this could be one explanation for the difference in BMI in men. It is well known that women often use smoking to control weight, but weight regulation does not seem to be a common reason for using smokeless tobacco [32,33].

One question is whether the statistical power was adequate to detect a relationship between snuff and incidence of CVD. Bolinder et al [8] reported that the age-adjusted RR of CVD was approximately 1.4 (95% CI: 1.2–1.6) for snuff users compared to non-users. If the risk associated with snuff had been of that magnitude, our probability of detecting a significant difference in incidence of MI would be about 75%. The statistical power for a combined endpoint of MI and stroke in the present study would be about 90%. We therefore believe that the statistical power of our study was adequate.

The MDC cohort was used in the present study. The participation rate was low in this cohort, approximately 40% [15]. However, when comparing the present cohort with results from a health survey based on a mailed questionnaire to subjects living in the city of Malmö 1994, in which the participation rates were 80%, it has been shown that the present cohort is fairly representative with respect to e.g., smoking habits and BMI [16]. The mortality rates in the study by Manjer et al were, however, higher among non-participants.

The results from the present study are similar to what has been reported from other population-based studies [2-4,6,10,12,13] and in contrast to some other Swedish studies [7,8]. As use of snuff historically has been closely associated to inferior socioeconomic position, it cannot be ruled out that the differences between the present study and the results of males by Bolinder et al [8] could be explained by differences in study populations and time periods. Ramstrom et al, [29] stated that people who stop smoking often start using snuff and reported that 58% used snuff as an aid to stop smoking. Furthermore, many continued to use snuff after smoking cessation. In the study by Huhtasaari et al, 50% of snuff users were former smokers [12]. In this present study we also found 57% snuff users among male ex-smokers. This fact sometimes causes frustration in caregivers and public health professionals. Whether snuff should be used as a device to stop smoking is intensively debated in Sweden. On one hand, there is no doubt that the health hazard of snuff is considerably smaller than that of smoking. On the other hand, there are several other nicotine based products for tobacco cessation and we cannot exclude the possibility that snuff could increase the risk of cancer [27,28]. In our opinion, more studies are needed to fully clarify the effects of snuff, especially in women, during pregnancy and in older individuals. It is important that information about snuff given to patients and the general public is accurate and reliable. All findings should be reported in order to increase the knowledge about the health effects of snuff.

## Conclusion

Even though some life-style risk factors seem to be more prevalent in snuff-users, the present study does not support any independent relationship between snuff and incidence of MI or stroke in men.

## Competing interests

The authors declare that they have no competing interests.

## Authors' contributions

EJ participated in the design of the study, performed the statistical analysis and drafted the manuscript. BH conceived of the study, participated in its design and revised the manuscript critically. All authors read and approved the final manuscript.

## Pre-publication history

The pre-publication history for this paper can be accessed here:


